# Overexpression of microRNAs miR-25-3p, miR-185-5p and miR-132-3p in Late Onset Fetal Growth Restriction, Validation of Results and Study of the Biochemical Pathways Involved

**DOI:** 10.3390/ijms23010293

**Published:** 2021-12-28

**Authors:** Gabriela Loscalzo, Julia Scheel, José Santiago Ibañez-Cabellos, Eva García-Lopez, Shailendra Gupta, José Luis García-Gimenez, Salvador Mena-Mollá, Alfredo Perales-Marín, José Morales-Roselló

**Affiliations:** 1 Instituto de Investigación Sanitaria La Fe, 46026 Valencia, Spain; Perales_alf@gva.es (A.P.-M.); jose.morales@uv.es (J.M.-R.); 2 Department of Obstetrics and Gynecology, Hospital Universitario y Politécnico La Fe, 46026 Valencia, Spain; 3 Department of Systems Biology and Bioinformatics, University Rostock, 18055 Rostock, Germany; shailendra.gupta@uni-rostock.de; 4 EpiDisease S.L, Parc Científic, University of Valencia, 46980 Paterna, Spain; j.santiago.ibanez@epidisease.com (J.S.I.-C.); eva.garcia@epidisease.com (E.G.-L.); j.luis.garcia@epidisease.com (J.L.G.-G.); salvador.mena@uv.es (S.M.-M.); 5 Consortium Center for Biomedical Network Research on Rare Diseases (CIBERER), Carrer d’Alvaro de Bazan, 10, 46010 Valencia, Spain; 6 Institute of Health Carlos III, Biomedical Research Institute INCLIVA, 46010 Valencia, Spain; 7 Department of Physiology, School of Medicine and Dentistry, University of Valencia, 46010 Valencia, Spain; 8 Department of Pediatrics, Obstetrics and Gynecology, School of Medicine and Dentistry, University of Valencia, 46010 Valencia, Spain

**Keywords:** late-onset fetal growth restriction, miRNA, network analysis, bioinformatics, fetal health, cerebroplacental ratio, miRNA-25-3p, miRNA185-5p, miRNA132-3p

## Abstract

In a prospective study, 48 fetuses were evaluated with Doppler ultrasound after 34 weeks and classified, according to the cerebroplacental ratio (CPR) and estimated fetal weight (EFW), into fetuses with normal growth and fetuses with late-onset fetal growth restriction (LO-FGR). Overexpression of miRNAs from neonatal cord blood belonging to LO-FGR fetuses, was validated by real-time PCR. In addition, functional characterization of overexpressed miRNAs was performed by analyzing overrepresented pathways, gene ontologies, and prioritization of synergistically working miRNAs. Three miRNAs: miR-25-3p, miR-185-5p and miR-132-3p, were significantly overexpressed in cord blood of LO-FGR fetuses. Pathway and gene ontology analysis revealed over-representation of certain molecular pathways associated with cardiac development and neuron death. In addition, prioritization of synergistically working miRNAs highlighted the importance of miR-185-5p and miR-25-3p in cholesterol efflux and starvation responses associated with LO-FGR phenotypes. Evaluation of miR-25-3p; miR-132-3p and miR-185-5p might serve as molecular biomarkers for the diagnosis and management of LO-FGR; improving the understanding of its influence on adult disease.

## 1. Introduction

Late-onset fetal growth restriction (LO-FGR) is characterized by an imbalance between fetal demands and placental supply and is associated with an increased incidence of perinatal complications and long-term neurodevelopmental sequelae. LO-FGR hypoxia tends to be subtle. Consequently, Doppler ultrasound alone or combined with serological markers has failed to accurately predict adverse perinatal outcome (APO), making the search for new markers a crucial issue in fetal medicine [1,2,3,4,5].

In the recent years an emerging interest for epigenetics has been developed, as this mechanism has been shown to participate in the surveillance of cellular gene expression, playing a role in the development of different disorders and diseases [6,7,8]. Currently, three epigenetic mechanisms have been well described: DNA methylation, histone modifications, and miRNAs transcription [8]. We particularly focused on the latter, as these molecules (small noncoding RNAs, 20–24 nucleotides long) can be secreted as exosome microparticles into the blood stream in order to participate in the mechanisms of cellular signaling. Interestingly, miRNAs can regulate cellular gene expression at the post-transcriptional level by targeting mRNAs to transiently block translation or degrade the mRNA [9,10,11]. Moreover, many miRNAs can regulate clusters of genes, controlled by the same promoters to work in a synergistic manner, and many can be expressed during pregnancy [12]. Of note, some miRNAs are essential in placental development [13] and can even contribute to fetal-mother signaling during embryo development [14]. Trophoblast cells in the placenta may also produce miRNAs that can be secreted into the maternal circulation [15]. Furthermore, the phenomenon of synergistic target regulation by cooperating miRNA pairs has been recently proposed, demonstrating the role of miRNAs in processes related to placental development directly related with FGR [16]. Finally, miRNA pair gene target triplex formation has also the potential to enhance both effectiveness and specificity of target repression [17,18,19]. The aim of this study was to validate the existence of differentially expressed miRNAs in cord blood of fetuses with late-onset FGR, and to investigate their biological function and possible role in the development of adult disease.

## 2. Results

The study included 48 fetuses examined with Doppler ultrasound after 32 weeks: 24 with normal growth and 24 with LO-FGR. In the LO-FGR group, the mean gestational age at scan, mean gestational age at delivery, mean CPR MoM (Cerebroplacental ratio multiples of the median) and mean birth weight (in grams) were 37.9 (SD 1.49), 38.4 (SD 1.51), 0.7 (SD 0.25), 2277 (SD 313, while in the normal group these values were 39.1 (SD 1.03), 39.7 (SD 1.16), 1.24 (SD 0.35) and 3178.5 (SD 443.5). Concerning maternal characteristics, the mean maternal age and mean body mass index (BMI) in LO-FGR fetuses were 31.54 (SD 5.07) and 22.19 (SD 2.93), respectively, while in the normal group these values were 34.00 (SD 4.09) and 23.74 (SD 3.33), respectively. Differences between groups in all cases were statistically significant.

### 2.1. Differential Expression of miRNAs in FGR

**Figure 1:**
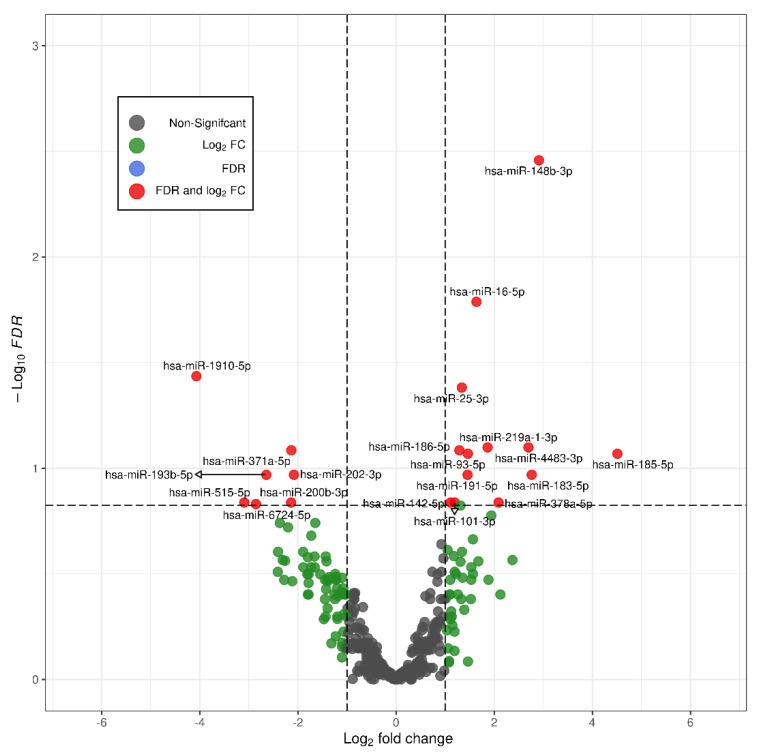
Volcano plot showing miRNA overexpression in LO-FGR fetuses. Vertical lines represent the log FC thresholds at −1 and 1, while the horizontal line represents the FDR threshold at 0.15.

In a previous study, we performed a high throughput smallRNA-sequencing study to explore differential expression of circulating miRNAs between LO-FGR and normal fetuses [20]. Although we found that in the LO-FGR group, miR-148b-3p, miR-16-5p and miR-25-3p were upregulated, and miR-1910-5p downregulated [20], we decided to select only miR-148b-3p and miR-25-3p for further analysis, as miR-16-5p had been associated with presence of hemolysis and miR-1910-5p was not related with any important biochemical pathway. This initial analysis with all the initial miRNAs involved is depicted in a volcano plot (Figure 1).

**Figure 2:**
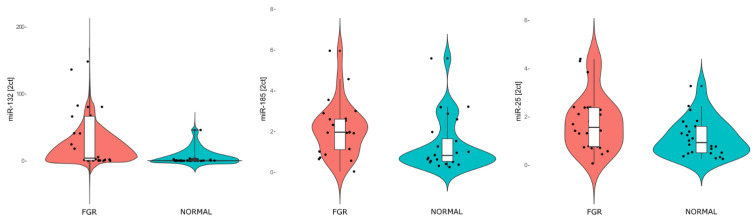
Violin plots representing PCR validation results of miR-132, miR-185 and miR-25. MiRNA results are expressed as 2^−ΔΔCt^.

When we now compared the expression levels using RT-qPCR [21], a significant overexpression of miR-185-5p and miR-25-3p was observed in the LO-FGR group (Mann–Whitney U-test). Conversely, miR-148b-3p, miR-183-5p, miR-193b-5p and miR-4483-3p did not show statistically significant differences (Figure 2 and Table 1).

### 2.2. Functional Analysis of Deregulated miRNA

**Figure 3:**
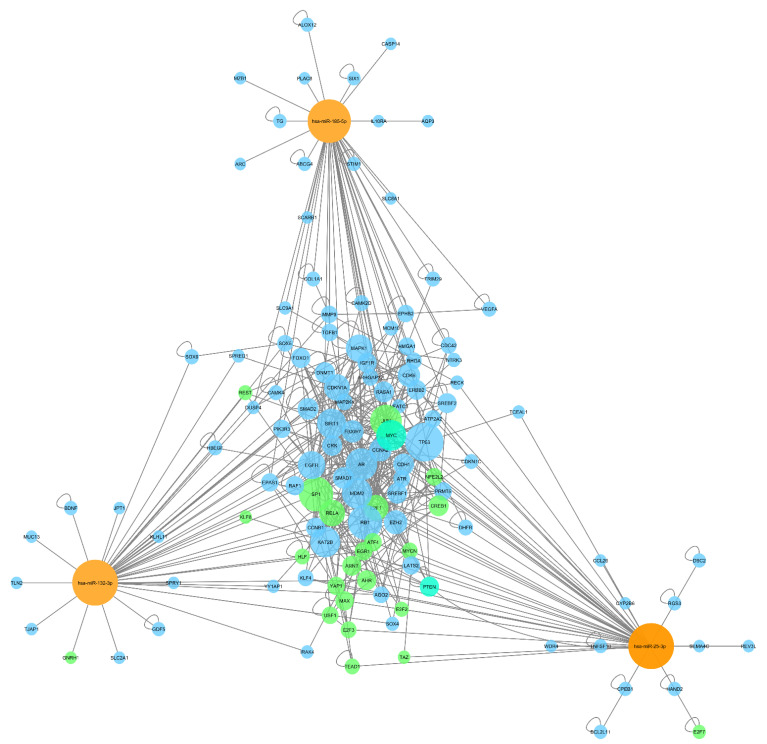
Interaction network of deregulated miRNAs including their target genes and transcription factors. Orange: miRNA. Blue: target gene. Green: transcription factors. Turquoise: transcription factor and target gene. Interactions between miRNA nodes and gene nodes indicate miRNA dependent repression of mRNA translation of the indicated gene. Interactions between genes indicate molecular interactions of their protein products. Node size indicates its degree (number of interactions).

The function of miRNAs in a biological sense, can be deferred from the biological function and processes their targets have and are involved in [22]. To this end the regulatory network of deregulated miRNA, their gene targets, and transcription factors was created. Deregulated miRNA gene target and transcription factor identification revealed 100 strong evidence miRNA-gene interactions, involving 98 unique gene targets and 26 (unique) transcription factors. The elements exhibit a high degree of interconnectedness (Figure 3). These targets were used for subsequent functional analysis.

To interpret the functional effect of deregulated miRNAs, both pathway enrichment analysis and Gene Ontology (GO) enrichment analysis, based on the regulatory network of deregulated miRNAs, were performed. Genes can have various functions, given the size of the regulatory network, enrichment analyses results indicate which biological processes and pathways are affected to a higher degree by specific molecular changes (i.e., by several genes within the regulatory network). This type of analysis was performed to reduce the complexity of required analysis, increasing functional explanatory potential [23]. Pathway enrichment analysis was performed in EnrichR (original results: https://maayanlab.cloud/Enrichr/enrich?dataset=d5dc3c01b3152f2af91f2bab49f38b5d; accessed on 03 August 2021) using their gene targets and transcription factors. Pathway enrichment analysis revealed a myriad of affected pathways. Kegg mainly shows cancer related pathways, apart from cellular senescence. Enriched Reactome 2016 pathways also indicate cellular senescence, in combination with cellular stress responses, and pathways involved in inflammation (FCERI), cell survival (NGF and TRKA), angiogenesis (PDGF) and general developmental biology (Table 2).

**Figure 4:**
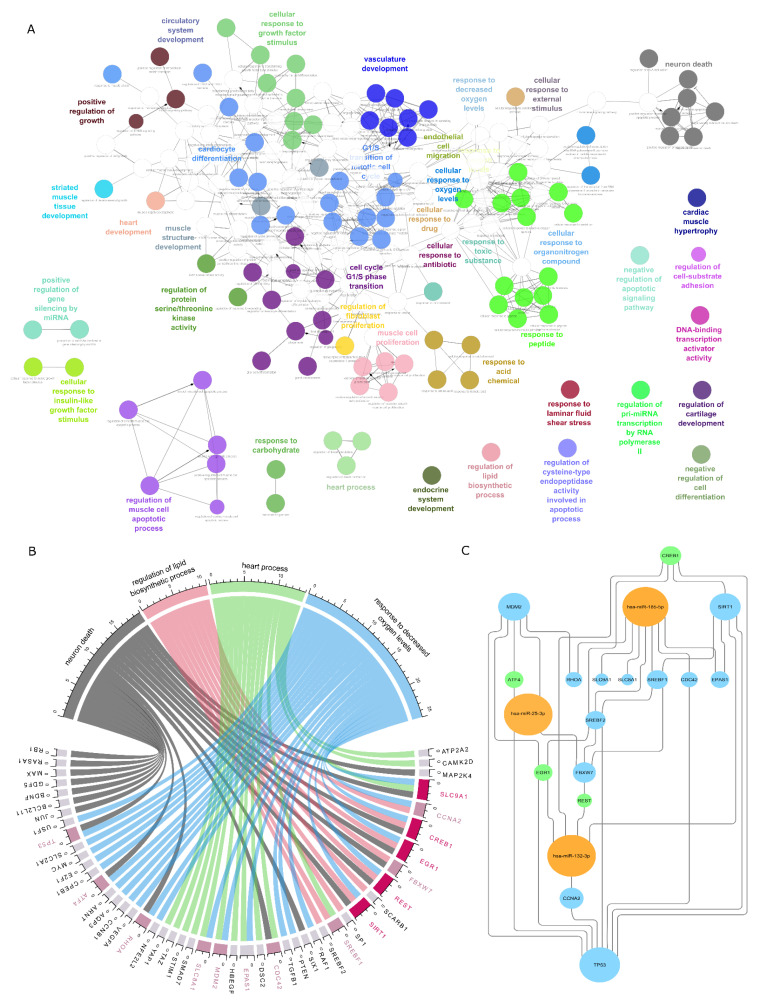
Functional analysis and regulatory module identification. ClueGO functional analysis based on the previous gene miRNA transcription factor network. Functionally related terms are grouped and visualized in the same color. Term similarity is indicated by node proximity (**A**). GO terms associated with phenotypes exhibited in FGR, “neuron death”, “regulation of lipid biosynthetic process”, “heart process”, and “response to decreased oxygen levels” including genes falling into these terms were used to filter the original regulatory network (**B**). Genes falling into more than one GO term are highlighted (pink). The filtered original network revealed regulatory elements consisting of deregulated miRNAs (orange), highly connected target genes (blue), and transcription factors (green), important for the manifestation of FGR phenotypes (**C**).

GO biological process functional analysis, based on the regulatory network (Figure 4), revealed 302 significantly (adj *p* < 0.001) overrepresented GO terms. The terms have been grouped according to GO term similarities. The largest groups contain terms related to cell cycle phases, cellular response to growth factor stimulus, vasculature and heart development, and neuron death (Figure 4A). Terms that are functionally related are located closer to each other. As some terms are redundant (i.e., “response to oxygen levels” and “response to low oxygen levels”, group terms are highlighted. To make the follow up interpretation less complex, we decided to focus on biological processes that have been associated with FGR. In particular processes related to “neuron death” [24], “regulation of lipid biosynthetic process” [25], “heart process” [26], “response to decreased oxygen levels” [27] are prominent in FGR and were selected as filters for the identification of regulatory elements, within the regulatory network (Figure 4B).

GO terms of interest including genes falling into these terms show which genes are involved in the seemingly unrelated processes (Figure 4B). In order to identify regulatory elements, we further filtered the regulatory network based on genes falling into more than one GO term of interest. These elements show a high amount of interconnectedness, further supporting a regulatory role in the phenotype presentation of FGR [28] (Figure 4C).

### 2.3. Interacting miRNA Triplex Formation

**Figure 5:**
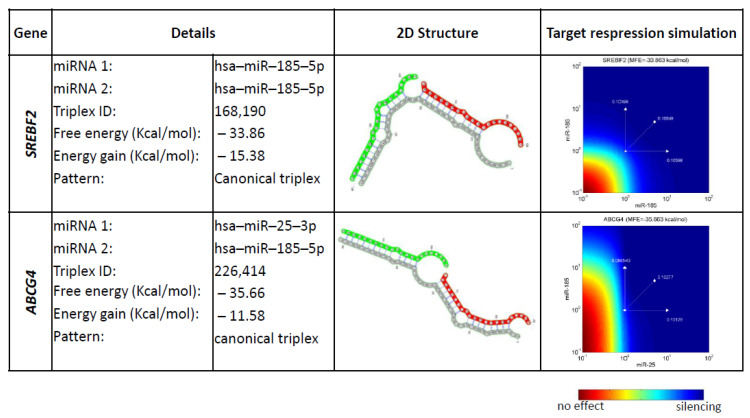
RNA triplexes formed by deregulated miRNAs and strong evidence target genes. 2D structure is shown in the third column. Repression efficiency is color coded with red having no effect and blue having a silencing effect.

Cooperatively working miRNAs consisting of miR-185-5p and miR-25-3p acting on the same target within the pool of experimentally validated strong-evidence gene targets, revealed two targets of interest SREBF2 and ABCG4. Both targets have relatively low free energy and energy gain scores (Figure 5). Prioritizing synergistically working miRNAs, genes not supported by strong experimental evidence were also included for consideration. Only genes affected by at least two miRNA pairs were considered. This revealed another five predicted repressed genes (Tables S1 and S2 in Supplementals S1), all of which are involved in fetal development [29,30,31,32,33], and have not ever been associated with FGR before. Three of these miRNA-target genes were associated with specific developmental processes, reinforcing the notion that miR-188-5p and miR-25-3p may have a specific role in FGR.

### 2.4. Limitation and Novelty

Sample Collection: Standard samples were used to perform the described experiments, to minimize invasive procedures. It is common practice to analyze blood pH at birth, which leads to a reduced volume within some samples. Hemolysis, insufficient volume (LO-FGR group) as well as the use of anticoagulants such as heparin in the sample tube are limiting factors that did not allow PCR reactions to be performed.

Novelty: In the previous pathway overrepresentation [20], the analysis was performed using DIANA-miRPath v3.0, which is based on miRNA-gene target identification using TarBase v7.0 [34]. In the current study miRTarBase v8.0 was used [22]. Further, traditional functional enrichment analysis does not consider triplex formations of miRNA couples repressing the same gene target cooperatively. This study further includes TriplexRNA to identify and prioritize targets that would usually be neglected.

## 3. Discussions

### 3.1. Intrauterine Environment as the Origin of Adult Disease

The influence of a suboptimal environment during the fetal period produces epigenetic changes that induce short- and long-term gene expression patterns [35,36] favoring, in many cases, susceptibility to certain chronic (metabolic, neurological, renal and cardiovascular) diseases [37,38,39,40]. This study attempts to clarify underlying epigenetic changes occurring in fetuses with late growth restriction and to highlight the importance of identifying fetuses at increased risk of APO.

In the presence of fetal hypoxia, the fetus will initially try to adapt to these conditions to ensure arrival of oxygen to vital areas such as the heart, brain, liver and kidneys [41]. This is reflected in the “brain sparing” phenomenon, in which the fetus redistributes the cardiac output to maximize oxygen and nutrient supply to the brain, with a decrease in the pulsatility index of the middle cerebral artery (MCA) (cerebral vasodilation) [42]. If the hypoxic condition persists, redistribution of blood flow can lead to altered structure and function of the vasculature, predisposing the fetus to late-onset diseases such as atherosclerosis, systemic hypertension, neurodegenerative diseases and chronic kidney disease [37,38,39,40] with major repercussions in adult life. The presence of this phenomenon in growth restricted fetuses is associated with smaller head circumferences and brain volumes at birth, and with later neurocognitive anomalies, resulting in poor learning and low academic results [43].

### 3.2. miR-25-3p

This miRNA has been involved in DNA damage, cell cycle regulation, cell proliferation and cell death. In addition, it has been implicated in the pathogenesis of multiple conditions such as acute diabetes, nephropathies, heart failure and myocardial affections [44,45,46,47,48]. Not surprisingly, their confirmed sites of action are the renal tubular epithelium, the endothelium of brain vessels and smooth muscle cells, again reflecting the association of growth restriction with alterations in the microvasculature [49,50].

### 3.3. miR-185-5p

Angiogenesis may play an important role in the development of LO-FGR [51]. Late growth restriction is frequently associated with a relatively normal placental function, which is otherwise insufficient to cope with the increasing fetal needs at the end of pregnancy [50]. This explains why LO-FGR occurs after 32 weeks, because at this gestational age, fetal nutritional requirements begin to increase, while placental function remains stable [3]. This is translated in a proportion of fetuses into a normal umbilical artery (UA) Doppler plus an abnormal MCA Doppler, reflecting a degree of hidden hypoxia [50]. The response to this hypoxia requires a high level of fetal coordination [51] with a spectrum of adaptive changes in gene expression. This process is mediated by angiogenic factors such as hypoxia-inducible factors (HIF) and vascular endothelial growth factor (VEGF) [52,53].

VEGF is an endothelial-specific factor, which promotes not only angiogenesis but also proliferation and migration of vascular proliferation and migration of vascular endothelial cells, increased vascular permeability and degeneration of the extracellular matrix [54]. More recent findings have revealed that VEGF has also direct effects on neuronal cells, as VEGF receptors are present on these cells. It was observed in animal models that impairment of VEGF expression, leads to the manifesting of signs of motor neuron degeneration [54]. Therefore, decreased VEGF levels would limit neural tissue perfusion and fetal neural protection, leading to neuronal disorders.

Not all tissues respond equally to hypoxic conditions. Each tissue presents different patterns of HIF expression, which is thought to be due to the tissue-specific expression of miRNAs [52]. MiR-185, has been identified as a modulator of vascular endothelial growth factor (VEGF) and its receptor (VEGFR) levels under the influence of hypoxia-inducible factors (HIF) [55]. In this study we observed overexpression of miR-185-5p which implies an inhibitory effect on VEGF action predisposing to alterations in the neural development and the vascular endothelium.

**Figure 6:**
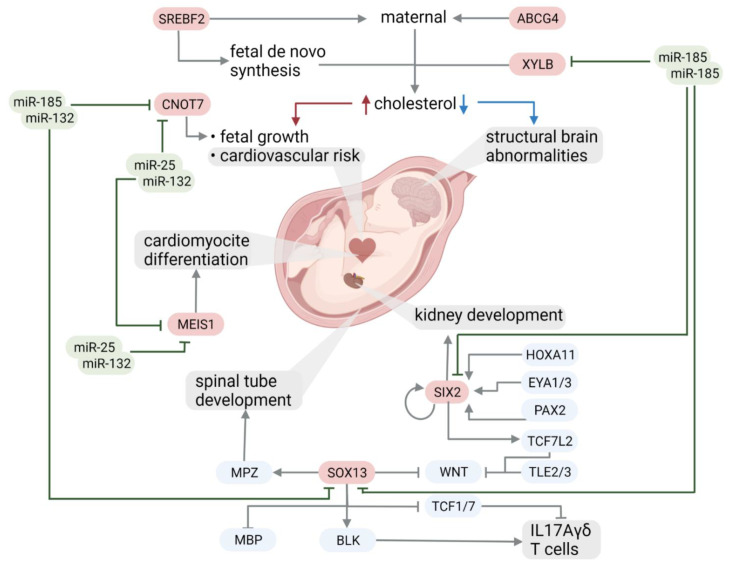
Targets of synergistically working miRNAs and their function. The created miRNA-gene target-transcription factor network, filtered for GO biological function terms neuron death, regulation of lipid biosynthetic process, heart process, and response to decreased oxygen levels revealed a highly interconnected subnetwork. To identify regulatory factors, this subnetwork was filtered for genes falling into more than one GO term of interest. The remaining genes still show high interconnectedness, including sharing interactions with SREBP-2, which has been indicated as a target of synergistically working miRNAs (Figure 4). These elements are thus indicated as regulatory elements for the exhibited phenotype and deserve further study as biomarkers and therapeutic targets for FGR [28].

The cardiovascular remodeling that occurs in growth-restricted fetuses results in structural changes that alter cardiovascular function [56]. Cardiac remodeling refers to the series of changes in the structure and function of the heart that can be seen early in fetal life in response to adverse conditions. During oxygen deprivation, the heart will try to cope with this shortage by changing its shape in order to ensure oxygen availability to the principal organs. Firstly, it may develop a more spherical shape to maintain systolic volume, and if the situation persists, cardiac hypertrophy develops to increase contractility [56,57,58]. Again, not surprisingly, both miR-132-3p and miR-25-3p target the MEIS1 gene (Figure 6), which is mainly involved in cardiomyocyte differentiation and cardiac muscle hypertrophy.

On the other hand, it is important to highlight the importance of cholesterol homeostasis in fetal development [59]. Cholesterol is an essential nutrient for fetal growth. The fetus has two ways to regulate cholesterol, one by passage through the placental barrier and the other by “de novo” fetal synthesis. Previous studies have shown that in FGR, reduced cholesterol is mostly due to a decrease in its synthesis [60], and this may be associated with future metabolic diseases, such as atherosclerosis [61,62,63].

Sterol response element binding protein (SREBP)-2 is a transcription factor, which plays a crucial role in the regulation of cholesterol metabolism by controlling the expression of genes related to de novo cholesterol biosynthesis and LDL uptake [60]. Studies on miR-185-5p have shown that its overexpression in liver cells, represses expression and level of SREBP-2, LDL uptake and HMG-CoA reductase activity [64,65]. All these changes are supported by the biological pathways we have described and more interestingly, it is the way in which they cooperate to exert their actions.

The analysis of synergistically working miRNAs highlighted another five predicted repressed genes all of which are involved in fetal development [29,30,31,32,33] and have not been associated with FGR before. SIX2 is involved in kidney development [29], and MEIS1 plays a role in oxidative phosphorylation in developing cardiomyocytes [30]. While the remaining two are associated with overall embryonic viability and placentation. SOX13 has been shown to regulate Hhex (hematopoietically expressed homeobox) and to repress Wnt/TCF signaling thus controlling Wnt/TCF signaling in the early embryo and more specifically differentiation of oligodendroglia in developing spinal tube (mbp, blk, il 17A) [31]. It further activates BLK, which activates IL17Agamma delta T cells and inhibits TCF1/7 which inhibits IL17A gamma delta T cells [66].

Finally, while CNOT7 is important for overall embryonic viability [67], oocyte to embryo development [32], and in general cell proliferation, invasion, and migration [68]. The latter being crucial to healthy placentation [69]. XYLB (xylulokinase) represents a key metabolic regulator in both glucose metabolism and lipogenesis [33] (Figure 6).

### 3.4. miR-132-3p

This miRNA was included in the analysis for its importance in controlling neuronal function [70,71,72]. In addition, we should mention that miR-132-3p, which arises from the miR-212/132 cluster family, has an important role in carcinogenesis of solid tumors [73] and is related with different abnormal conditions in the hepatic [74] and adipose tissue [75]. However, as indicated, its pivotal role relates with the regulation of several signaling pathways in neuronal development (neuronal morphogenesis and synaptic plasticity) [70,71,72] and in the regulation of cardiac hypertrophy and autophagy in cardiomyocytes [76,77].

## 4. Materials and Methods

### 4.1. Patient Recruitment

This was a prospective study in which 75 pregnant women were recruited and samples collected at the public tertiary maternity ward of La Fe Hospital between 2019 and 2020. The final results were obtained from 48 samples, as 18 samples failed quality control and were excluded due to the presence of hemolysis in the samples—and 9 samples did not amplify in RT-qPCR and were excluded.

Ineligibility criteria included maternal age less than 18, absence of written consent, pregnancies with fetal malformations in any of the three trimesters and cases delivered out of La Fe Hospital.

All patients were recruited during routine follow-up ultrasonography, performed at 32–41 weeks gestation, using General Electric Voluson^®^ (E8/E6/730) ultrasound machines (General Electric Healthcare, Madrid, Spain) with 2–8 MHz convex probes. Examinations included evaluation of EFW local centiles [78] and Doppler parameters following international guidelines [79], such as the UA pulsatility index, the MCA pulsatility index and their ratio, the cerebroplacental ratio (CPR) [17].

Based on these parameters, pregnant women were classified into two groups. The first included fetuses with normal growth (NG) (EFW > 10thcentile or EFW at the 3rd–10th centile plus a normal CPR), and the second (LO-FGR) included fetuses with abnormal growth (EFW < 3rd centile or EFW at the 3rd–10th centile plus an abnormal CPR) [79,80]. Gestational age was in all cases corrected by means of the crown-rump length in the first trimester [81]. Finally, maternal data, such as gravidity (number of times a woman had been pregnant regardless of the outcome), parity (number of times a woman had given birth to a fetus with a gestational age of 24 weeks or more, regardless of whether the infant was born alive or stillborn), weight, height, mode of delivery and destination of the newborn (maternal ward or neonatology) were collected from hospital files.

All patients were followed up until labor in order to obtain cord and maternal blood samples immediately after delivery. After birth, plasma samples were collected from the fetal umbilical vein and maternal peripheral blood was collected in EDTA tubes and centrifuged at 3500 rpm for 10–15 min to obtain plasma. Once obtained, each sample was stored at −80 °C until small RNA extraction (maternal plasma was stored for future research). Fetal blood plasma (500 μL) was used to isolate total cell–free RNA (including miRNAs) using the miRNeasy Serum/Plasma kit (Qiagen, Carlsbad, CA, USA) following the manufacturer’s protocol.

As above indicated, the first analysis we performed [20] was high-throughput small RNA sequencing to explore the differential expression of circulating miRNAs in LO-FGR (Figure 1). In the current study, these miRNAs were validated by PCR. Finally, once the results of the RT-qPCR validation were obtained, a functional characterization of overexpressed miRNAs was performed.

The Six miRNAs currently validated by PCR were those miRNAs that showed high differential expression in the small RNA sequencing: mir-25-3p, miR 148-3p, miR-185-5p, miR-183-5p, miR-4483-3p and miR-193b-5p [20], according to the following criteria: FDR < 0.05 or FDR < 0.15 and logFC ≥ 2.5 in absolute values. This has been depicted in the volcano plot (Enhanced Volcano R-Pachage v 4.1.2). In addition, although we did not obtain a differential expression of miR-132-3p in the initial genetic analysis [20], we studied miR–132-3p in the validation and pathways analysis for its important role in maintaining and promoting neuronal activity according to earlier references [70,71,72].

The study protocol was approved by the local ethics committee (Comité de Ética de Investigación con Medicamentos (CEIm) Hospital Universitario y Politécnico La Fe, Reference code 2016/453). All women gave written informed consent to participate in the study.

### 4.2. Quantitative Reverse Transcription Polymerase Chain Reaction (RT-qPCR) Validation in Umbilical Cord Samples

RT-qPCR experiment was performed to confirm the eight miRNAs obtained with the NGR analysis. A total of 48 umbilical cord samples (24 normal growth fetuses and 24 late-onset FGR), were tested. Reverse transcription reactions (RT-PCR), were performed using the TaqMan^®^ miRNA Reverse Transcription kit (Part No. 4366596, Applied Biosystems, Inc., CA, USA) and miRNA-specific stem-loop primers (Part No. 4366596, Applied Biosystems, Inc., Carlsbad, CA, USA) [miR-148b-3p (Assay ID 000471), miR-25-3p (Assay ID 000403), miR-185-5p (Assay ID 002271), miR-183-5p (Assay ID 002269), miR–4483–3p (Assay ID 461903_mat), miR-193b-5p (Assay ID 002366), miR-132-3p (Assay ID 000457), and hsa-miR-191-5p (Assay ID 002299) and 100 ng of input cell-free RNA in a 20 µL RT reaction. The RT-PCR conditions were 16 °C for 30 min, followed by 42 °C for 30 min, and a final inactivation step for 5 min at 85 °C.

The Real-time PCR reactions were performed in triplicate, in scaled–down 10 µL reaction volumes using 5 µL TaqMan^®^ 2X Universal PCR Master Mix with No UNG (Applied Biosystems, Inc., Carlsbad, CA, USA), 0.5 µL TaqMan^®^ Small RNA assay (20X) (Applied Biosystems, Inc., Carlsbad, CA, USA), 3.5 µL of nuclease free water and 1 µL of RT product. The PCR conditions were 95 °C for 10 min, followed by 45 cycles at 95 °C for 15 s and 60 °C for 1 min. The hsa-miR-191-5p served as an endogenous control for normalization. The relative quantification of miRNAs was calculated by the 2^−ΔΔCT^ method [21].

### 4.3. Gene Target Identification and Network Analysis

Target genes of validated miRNAs by RT-qPCR were identified using miRTarBase v8.0 [22]. To minimize false positives only experimentally validated strong-evidence miRNA-gene target pairs were considered. Literature-curated transcription factors and miRNA pairs were extracted from TransmiR [81]. miRNA, target gene, and transcription factor interactions were visualized in Cytoscape v3.8.2, while additional interactions between gene products were added using BisoGenet [82,83] (Tables S3 and S4 in Supplementals S2). Topological analysis (degree) was performed using the Cytoscape network analyzer.

### 4.4. Overrepresentation and Functional Analysis

The analysis of overrepresented gene ontology (GO) terms and pathways associated with the target genes, was performed using Cytoscape ClueGO plugin and EnrichR webtool [84,85]. Ontology and pathway GO terms with an adjusted *p*-value < 0.05, and <0.001 in ClueGO were considered significantly overrepresented. ClueGO results were corrected using Bonferroni step down correction and include term fusion. GO terms of interest with genes in common were visualized using the R packages GOPlot [86], gridExtra [87] (R package version 2.3), ggplot2 [88], R ColorBrewer [89]. RColorBrewer: ColorBrewer Palettes (R package version 1.1.2), and circlize [90] (circlize implements and enhances circular visualization in R Bioinformatics). In addition, gene expression networks were built in Cytoscape [82]. The KEGG, Reactome and WikiPathways databases, as well as the cell and tissue expression Human Gene Atlas (BioGPS) and Tissue Expression ArchS4 databases were used to obtain specific gene annotations [19,91,92,93] (Table S5 in Supplementals S3).

### 4.5. Synergistically Working miRNA

Interacting miRNA couples, synergistically acting on a gene target, as well as miRNA couple, gene 2D structure, and surface plots were identified using the triplex RNA web interface (https://triplexrna.org/, accessed on 07 July 2021) [19].

### 4.6. Statistical Analysis

Analysis was conducted on normalized data. Fold-change (FC) expression differences were obtained using the delta-delta CT method (2^−ΔΔCT^ or 2^−ddCt^) and Student’s *t*-test *p*-values (<0.05) were used as thresholds to identify deregulated miRNA across the two groups.

## 5. Clinical Significance

LO-FGR is often underestimated due to its subtle presentation. In this study we have described only some of the epigenetic mechanisms that might be activated under hypoxia. Overexpression of miRNAs 25-3p, 132-3p and 185-5p has shown a strong association with molecular pathways involved in the pathophysiology of growth restriction. Given these results of cooperating miRNAs repressing both cholesterol synthesis and efflux to the fetus, and the role of cholesterol as a factor in fetal brain development and cardiovascular risk, a closer look at the role of miR 185-5p and miR-25-3p as well as SREBF2 and ABCG4 as possible therapeutic targets should be taken in the future. We further identified a regulatory module consisting of the identified miRNAs, selected target genes and transcription factors. These are biomarker candidates, as their manipulation may ameliorate the phenotype exhibited here. Future studies are needed to confirm these markers in maternal blood and allow us to use them as tools in the prevention, diagnosis and/or therapy of this condition.

## Figures and Tables

**Table 1:** RT-qPCR validation of miRNAs in FGR and normal fetuses. miR-132-3p was also included in the analysis according to previous references. Only miR-185-5p, miR-25-3p and miR-132-3p showed statistically significant differences. Values are expressed in 2 ^−ΔΔCt^.

Expression Levels of the miRNAs in the Two Study Groups (Mean ± Standard Deviation)			
**MiRNAs**	**FGR**	**Normal**	***p***-**Value**
miR-132-3p	26.00 ± 33.78	1.08 ± 0.91	0.0002
miR-185-5p	1.80 ± 0.90	1.22 ± 0.95	0.03
miR-25-3p	1.76 ± 1.09	1.19 ± 0.82	0.05
miR-148b-3p	1.51 ± 0.80	1.11 ± 0.52	0.18 (NS)
miR-183-5p	1.23 ± 0.61	1.27 ± 1.64	0.24 (NS)
miR-193b-5p	1.53 ± 2.14	1.37 ± 1.55	0.18 (NS)

**Table 2:** The ten most significantly enriched Kegg 2021 human and Reactome 2016 pathways sorted by adjusted p-value, based on a deregulated miRNA gene target network.

Pathway	Adj *p*-Value
Kegg 2021 Human	
Pathways in cancer	6.709 × 10^−27^
Bladder cancer	1.085 × 10^−23^
Prostate cancer	1.085 × 10^−23^
Cellular senescence	1.932 × 10^−22^
Human T-cell leukemia virus 1 infection	5.516 × 10^−22^
Pancreatic cancer	6.030 × 10^−21^
Glioma	2.045 × 10^−19^
Hepatitis B	2.045 × 10^−19^
Human cytomegalovirus infection	3.192 × 10^−19^
MicroRNAs in cancer	1.116 × 10^−18^
**Reactome 2016**	
Cellular responses to stress Homo sapiens R-HSA-2262752	3.128 × 10^−13^
Cellular Senescence Homo Sapiens R-HSA-2559583	7.922 × 10^−13^
Signal Transduction Homo Sapiens R-HSA-162582	9.454 × 10^−13^
Fc epsilon receptor (FCERI) signaling Homo sapiens R-HSA-2454202	4.211 × 10^−11^
Signaling by NGF Homo sapiens R-HSA-166520	4.211 × 10^−11^
Signaling by EGFR Homo sapiens R-HSA-177929	4.704 × 10^−11^
NGF signaling via TRKA from the plasma membrane Homo sapiens R-HSA-187037	1.014 × 10^−10^
Downstream signal transduction Homo sapiens R-HSA-186763	1.937 × 10^−10^
Signaling by PDGF Homo sapiens R-HSA-186797	4.611 × 10^−10^
Developmental Biology Homo Sapiens R-HSA-1266738	4.611 × 10^−10^

## Data Availability

Data available in a publicly accessible repository that does not issue DOIs (FAIRDOMHub) Publicly available datasets were analyzed in this study. This data can be found here: https://fairdomhub.org/projects/275.

## Supplementary Materials

The following supporting information can be downloaded at: https://www.mdpi.com/article/10.3390/ijms23010293/s1.
