# Supplementary material for: Overexpression of microRNAs miR-25-3p, miR-185-5p and miR-132-3p in Late Onset Fetal Growth Restriction, Validation of Results and Study of the Biochemical Pathways Involved
Source: Int J Mol Sci. 2021 Dec 28;23(1):293. doi: 10.3390/ijms23010293 (PMC8745308; doi:10.3390/ijms23010293)
Supplement: Supplementary file 1 [file ijms-23-00293-s001.zip › Supplementals 1_ TriplexRNA and Table of genes invovled in more than one GO term of interest.pdf]

Synergistically working miRNAs

Cooperatively working miRNAs have only fairly recently been able to be computed (Schmitz et al, 2014). Traditional functional analysis pipelines such as EnrichR and ClueGO do not consider these targets, yet (Chen et al, 2003, Bindea et al, 2009).

To improve functional interpretation of deregulated miRNAs, predicted gene targets were included for consideration. Only genes targeted by at least two synergistically working miRNA pairs were considered. The lower free energy and energy gain, the more likely these miRNAs will pair triplexes with their target.

Table S1. Predicted miRNA pair - gene triplexes. MiRNA that have the ability to repress a gene in a synergistic manner. Free energy and energy gain of said triplex formation indicate both likelihood and efficacy of triplex formation.

Gene	miRNA1	miRNA2	Free Energy [kcal/mol]	Energy gain [kcal/mol]	Pattern
SIX2	hsa-miR-185	hsa-miR-185	-35.26	-16.88	Canonical triplex
	hsa-miR-185	hsa-miR-185	-34.16	-15.98	Canonical triplex
MEIS1	hsa-miR-25	hsa-miR-25	-23.16	-9.08	target self-complementarity
	hsa-miR-25	hsa-miR-132	-22.26	-9.78	Canonical triplex
SOX13	hsa-miR-132	hsa-miR-185	-34.06	-15.18	Canonical triplex
	hsa-miR-185	hsa-miR-185	-38.56	-18.08	Canonical triplex
CNOT7	hsa-miR-25	hsa-miR-132	-24.46	-6.28	miRNA duplex
	hsa-miR-132	hsa-miR-185	-28.26	-11.48	Canonical triplex
XYLB	hsa-miR-185	hsa-miR-185	-33.76	-15.28	miRNA self-complementarity
	hsa-miR-185	hsa-miR-185	-31.46	-15.28	miRNA self-complementarity

Regulatory elements of the FGR phenotype

Functional analysis of the miRNA - target gene - transcription factor regulatory network revealed a myriad of overrepresented biological process GO terms. These were selected for processes known to be deregulated in FGR. To further filter for regulatory elements, only genes falling into more than one GO term were

considered (Table S2). These elements were then used to filter the existing miRNA - target gene - transcription factor network. As seen in Figure 4C (main manuscript) these targets show high interconnectedness indicating a regulatory role (https://www.frontiersin.org/articles/10.3389/fgene.2019.01120/full) considering the selected biological processes (heart process, response to decreased oxygen levels, neuron death, regulation of lipid biosynthetic process).

Table S2. Functional analysis of the miRNA - target gene - transcription factor regulatory network overrepresented biological process GO terms.

Gene	No° processes	Processes	miRNA	Degree
SLC9A1	3	Heart process, response to decreased oxygen levels, neuron death	hsa-miR-185-5p	2
REST	3	Response to decreased oxygen levels, regulation of lipid biosynthetic process, neuron death	hsa-miR-132-3p	4
SIRT1	3	response to decreased oxygen levels, regulation of lipid biosynthetic process, neuron death	hsa-miR-132-3p	22
CREB1	3	Response to decreased oxygen, regulation of lipid biosynthetic process, neuron death	hsa-miR-132-3p	11
EGR1	3	Response to decreased oxygen, regulation of lipid biosynthetic process, neuron death	hsa-miR-132-3p	8
FBXW7	2	regulation of lipid biosynthetic process, neuron death	hsa-miR-25-3p	13
SLC8A1	2	Response to decreased oxygen levels, heart process	hsa-miR-185-5p	1
RHOA	2	response to decreased oxygen levels, neuron death	hsa-miR-185-5p	5
CCNA2	2	Response to decreased oxygen levels, regulation of lipid biosynthetic process	hsa-miR-132-3p	14
SREBF1	2	Heart process, regulation of lipid biosynthetic process	hsa-miR-185-5p	11
CDC42	2	Heart process, neuron death	hsa-miR-185-5p	6

EPAS1	2	Response to decreased oxygen levels, heart process	hsa-miR-185-5p	8
MDM2	2	Response to decreased oxygen levels, heart process	hsa-miR-25-3p	23
ATF4	2	Response to decreased oxygen levels, neuron death	hsa-miR-25-3p	8
TP53	2	Response to decreased oxygen levels, neuron death	hsa-miR-25-3p	33
